# Molecular immune monitoring in kidney transplant rejection: a state-of-the-art review

**DOI:** 10.3389/fimmu.2023.1206929

**Published:** 2023-08-22

**Authors:** Wiwat Chancharoenthana, Opas Traitanon, Asada Leelahavanichkul, Adis Tasanarong

**Affiliations:** ^1^ Department of Clinical Tropical Medicine, Faculty of Tropical Medicine, Mahidol University, Bangkok, Thailand; ^2^ Tropical Immunology and Translational Research Unit (TITRU), Department of Clinical Tropical Medicine, Faculty of Tropical Medicine, Mahidol University, Bangkok, Thailand; ^3^ Thammasat Multi-Organ Transplant Center, Thammasat University Hospital, Faculty of Medicine, Thammasat University, Pathumthani, Thailand; ^4^ Division of Nephrology, Department of Medicine, Faculty of Medicine, Thammasat University, Pathumthani, Thailand; ^5^ Center of Excellence on Translational Research in Inflammation and Immunology (CETRII), Department of Microbiology, Chulalongkorn University, Bangkok, Thailand; ^6^ Department of Microbiology, Faculty of Medicine, Chulalongkorn University, Bangkok, Thailand

**Keywords:** chemokine, donor-derived cell-free DNA, exosomes, extracellular vesicles, MicroRNAs, molecular immune monitoring, nucleosome, transcriptomics

## Abstract

Although current regimens of immunosuppressive drugs are effective in renal transplant recipients, long-term renal allograft outcomes remain suboptimal. For many years, the diagnosis of renal allograft rejection and of several causes of renal allograft dysfunction, such as chronic subclinical inflammation and infection, was mostly based on renal allograft biopsy, which is not only invasive but also possibly performed too late for proper management. In addition, certain allograft dysfunctions are difficult to differentiate from renal histology due to their similar pathogenesis and immune responses. As such, non-invasive assays and biomarkers may be more beneficial than conventional renal biopsy for enhancing graft survival and optimizing immunosuppressive drug regimens during long-term care. This paper discusses recent biomarker candidates, including donor-derived cell-free DNA, transcriptomics, microRNAs, exosomes (or other extracellular vesicles), urine chemokines, and nucleosomes, that show high potential for clinical use in determining the prognosis of long-term outcomes of kidney transplantation, along with their limitations.

## 1 Introduction

A kidney transplant is typically the best option for patients with end-stage renal disease (ESRD). Kidney transplant (KT) recipients have a life expectancy that is more than double that of people on dialysis, and they also have a significant improvement in their quality of life (1). Furthermore, kidney transplantation is the most cost-effective long-term therapy for people with ESRD. Treatment developments have led to a steady decline in long-term allograft failure over the past 15 years: the kidney allograft failure rates five years post-transplantation in recipients receiving kidneys from deceased donors (DD) and live donors (LD) dropped to 14% and 9%, respectively in the periods from 1996 to 2012. The long-term survival of DD recipients has increased from 8.2 years (between 1995 and 1999) to 11.7 years (between 2014 and 2017) (2). Data from the National Kidney Transplantation Registry of Thailand in 2019 revealed the renal allograft survival rates at one, five, and ten years for DD recipients were 95.9%, 78.5%, and 58.5%, respectively. Meanwhile LD recipients showed a better renal allograft outcome than that of DD recipients (renal allograft survival rates were 98.2%, 92.6%, and 77.8%, respectively) (3). Of note, the leading causes of early graft failure within five years were rejection (56%) and interstitial fibrosis and tubular atrophy (IF/TA) (22%) followed by vascular or urologic complications (11%). IF/TA were the leading causes of late allograft failure (46.3%), followed by rejection (33%) and recurrent glomerular diseases (9%) (3). Thus, the major etiology of returning to dialysis in KT recipients is still dialysis reinstitution due to the failure of the renal allograft (3, 4). Despite advances in immunosuppressants and the management of acute kidney allograft rejection, a better understanding of several aspects of kidney transplantation is still needed, especially to improve long-term renal allograft survival. As such, donor characteristics and recipient variables (age, gender, dialysis vintage, and comorbidity), immunosuppressive drug monitoring, and immunological aspects such as human leucocyte antigen (HLA) mismatch, delayed graft function (DGF), cold ischemia period, and acute rejection during the first year of transplantation, have all been linked to long-term graft survival (5–8). Currently, several noninvasive biomarkers, including molecules, proteins, and immune responses, in combination or as single factors, have been developed to identify the risk of allograft rejection (9–12).

In response to the growing use of minimally invasive biomarkers in clinical transplantation, the Banff Minimally Invasive Biomarkers Working Group was established in early 2021 to examine the application of biomarkers in the diagnosis and categorization of renal allograft rejection. In the Banff 2005 and 2017 classification, donor-specific antibody (DSA) was introduced as a criterion for antibody-mediated rejection (AMR) (13, 14), and the classification of AMR and T cell-mediated rejection (TCMR) was greatly modified in the Banff 2019 classification (15). Currently, non-DSA biomarkers are mentioned in the Banff classification as screening tests to: *i)* rule out rejection, *ii)* expedite a confirmatory renal biopsy, or *iii)* directly diagnose rejection, either alone or in conjunction with histology (15, 16). Hence, the ideal biomarkers for diagnosis of allograft rejection should be able to distinguish rejection from non-rejection, be specific to rejection, replace biopsies or add information to the biopsy, and lastly, demonstrate prognostic value. The biomarker should also be able to discriminate between AMR and TCMR, which are induced through different immunopathogenic mechanisms. Several biomarkers include donor-derived cell-free deoxyribonucleic acid (dd-cfDNA), transcriptomic patterns, micro ribonucleic acids (microRNAs), exosomes, extracellular vesicles, chemokines, and nucleosomes are mentioned.

Our aim in writing this review was to summarize the most current research regarding novel biomarkers in the kidney transplantation field in terms of allograft rejection and their relevance to outcomes. Currently, novel biomarker use can be classified into two categories as immunological biomarkers and non-immunological biomarkers. The immunological biomarkers identify immune dysfunctions ranging from subclinical to overt rejection, whereas the non-immunological biomarkers indicate adverse transplant outcomes, such as delayed graft function, cardiovascular events, infection, and cancer, in which immune dysfunction is not the primary abnormality. Accordingly, although the non-immunological testing is necessary for long-term renal allograft outcomes, these biomarkers are outside the scope of this review.

## 2 Pathophysiology of renal allograft rejection

### 2.1 T cell-mediated rejection

**Figure 1:**
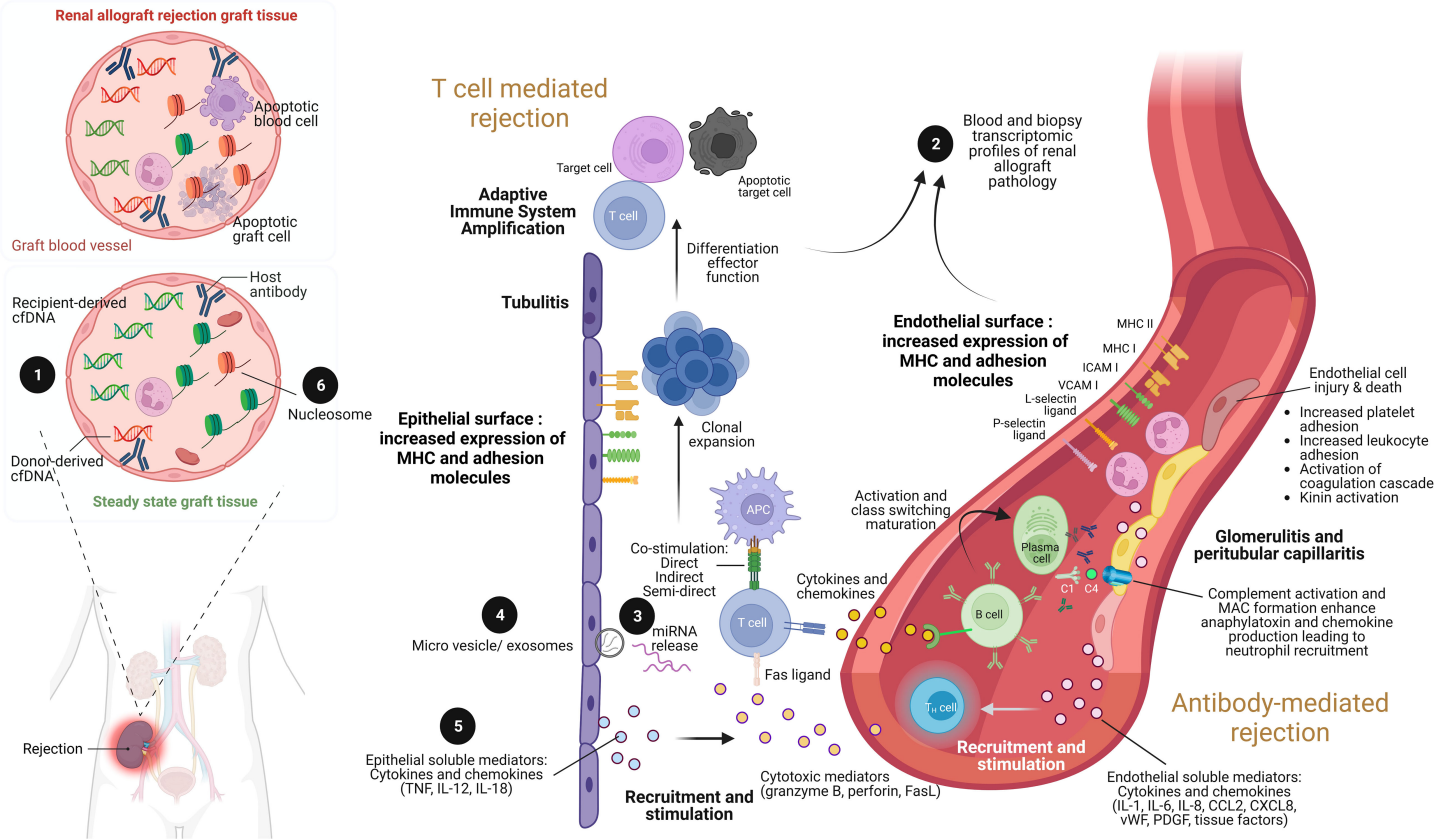
The illustration of renal allograft rejection and the application of biomolecular biomarkers from immunological pathogenesis. While the main pathogenesis of acute cellular mediated rejection (TCMR) is epithelial cell injury enhancement leading to adaptive immune system amplification, acute antibody-mediated rejection (AMR) is endothelial cell injury through antibody and complement enhancement. As a result, the culprit pathologic characteristic of TCMR is tubulitis compared to glomerulitis and peritubular capillaritis in AMR. Both patterns can be concurrently found in severe combined TCMR and AMR case. During renal allograft rejection process, both innate and adaptive immune system are activated from the imbalance differentiation between donor and recipient cell-free deoxyribonucleic acid (cfDNA) molecules ❶ (left panel). The final products of both T cell and B cell activation can be detected their signals and cellular origin of either peripheral blood or renal allograft tissue by transcriptomic profiles ❷. Indeed, plenty of mediators are produced during overwhelming inflammatory process from both TCMR and AMR, including the production of miRNAs ❸ within extracellular vesicles and exosomes ❹, and soluble mediators (cytokines and chemokines) ❺. Interestingly, the epigenetic control of gene expression by circulating cell-free nucleosome may play as a crucial step of renal allograft rejection activation ❻. Imbalance between donor- and recipient-derived nucleosomes with histone alteration is currently postulated as one of pathogenesis in renal allograft rejection. ICAM, intracellular adhesion molecule; IL, interleukin; MHC, major histocompatibility complex; VCAM, vascular cell adhesion molecule; TNF, tumor necrosis factor. Picture is created by BioRender.com.

Both innate and adaptive immune response components contribute to T cell-mediated graft injury. As such, the damage-associated molecular patterns (DAMPs) that are released in response to the ischemia during the graft preparation are recognized by pattern recognition receptors (PRRs) of phagocytic cells of the innate immunity leading to the upregulation of costimulatory molecules and secretion of pro-inflammatory cytokines (17). Mismatched HLA epitopes on the graft are recognized subsequently by host T cell receptors via direct, indirect, and semi-direct pathways (**Figure 1**) and act in concert with innate immunity-derived stimuli to activate and expand recipient T cell clones with inflammatory or regulatory functions (17). The production and release of soluble mediators, including interleukin (IL)-15, IL-17, granzyme B, perforin, *Fas* ligand which is also known as tumor necrosis factor (TNF) ligand superfamily member 6, interferon (IFN)-γ, TNF, CXC-chemokine ligand (CXCL) 10, CC-chemokine ligand (CCL) 2, CCL3, CCL4, CCL5, and CX3CL1, potentiates the inflammatory injury that is the characteristics of acute allograft rejection (17). Then, the activated mononuclear cells accumulate in the renal interstitium, tubules, and, rarely, in the arteries of the graft (leading to endarteritis), whereas glomerulitis may occur in more severe cases of rejection and is accompanied by apoptosis of vascular endothelial cells and mesangiolysis.

Currently, the Banff classification (13) stratifies TCMR-induced graft injury into three classifications based on the presence of *i)* interstitial inflammation in the non-scarred area of the cortex, *ii)* tubulitis in cortical tubules within the non-scarred cortex, and *iii)* endarteritis (intimal and transmural arteritis with fibrinoid necrotic change) as well as medial smooth muscle necrosis with lymphocytic infiltration in the vessel (18). Despite the fact that TCMR normally responds rapidly to immunosuppressive drugs, persistent inflammation in the areas of IF/TA is frequently associated with sustained expression of gene transcripts characteristic of acute kidney injury and predicts progression to chronic-active TCMR (19, 20).

### 2.2 Antibody-mediated rejection

Antibody-mediated rejection (AMR) is the most severe and destructive form of immune-mediated graft injury which is observed in approximately 30% of all patients with rejection (21). As such, AMR may occur with or without TCMR and can be detected early or late in the transplantation process, ranging from acute AMR with rapid and severe graft injury to chronic AMR with progressive graft destruction (21). Recipient CD4^+^ T cells, which are activated by epitopes expressed on graft antigens, assist in the activation of graft-specific B cells, which is followed by class switching and affinity maturation; T cell assistance is mediated by costimulatory factors and receptors, including inducible T cell costimulator, CD40 ligand, CD80, and CD86 (22). These activated B cells generate plasmablasts and plasma cells that produce DSAs (**Figure 1**). It has been reported that 15% of KT recipients developed *de novo* DSAs over 4 years after transplantation, and graft survival at 10 years was diminished by 40% compared to patients without *de novo* DSAs due to chronically active AMR (23). Solid-phase assays can be used to detect DSAs and enable precise determination of alloreactivity, which is frequently directed against HLA class II epitopes but has also been observed against non-HLA targets such as type 1 angiotensin II receptor, perlecan, and collagen (24). The antibody against HLA is frequently initially circumscribed to mismatched epitopes expressed on the graft; however, repeated stimulation may enhance sensitization and broaden the epitope repertoire via intramolecular and intermolecular antigen spreading (epitope spreading) (25).

As highlighted by the Banff criteria, DSA binding to target epitopes expressed on the vascular endothelium led to acute microvascular injury that can be characterized by endothelial cell enlargement, vacuolization, loss of fenestrations, detachment from the basement membrane, and apoptosis (26). Mobilization of endothelial vesicles externalizes P-selectin, facilitates the binding of several cells at the site of injury, including platelets, neutrophils, macrophages, natural killer cells, and T cells, contributes to intimal arteritis which is a major characteristic of AMR injury (26). The formation of the membrane attack complex (C5b–C9), which exacerbates injury to the endothelium and other graft tissues (27), is triggered by the binding of the complement C1 complex to activate the classic complement pathway (**Figure 1**). Immunoglobulin subclasses 1 and 4 of the DSA are associated with enhanced C1 binding capacity and the degree of complement activation and may therefore determine the severity of the injury. Hence, C4d is frequently deposited at the site of complement activation, whereas C3a and C5a function as anaphylatoxins enhancing infiltration in the kidney with innate immune cells (neutrophils and mononuclear inflammatory cells) that exacerbate the injury. Additionally, the complement-independent pathways may also be involved in AMR (21). As such, AMR is currently classified as active, smoldering, or chronic mechanisms and either the smoldering or chronic AMR is frequently resistant to treatment. Although none of the current therapeutic interventions has shown promising results in AMR, removal of circulating antibodies by plasmapheresis with the concurrent intravenous immunoglobulin administration to downregulate B cell activity is currently a standard of care (21) with inadequately supported evidence. Moreover, proteasome inhibitors, C1q or C5 inhibitors, anti-CD20 biologics, and cleaving endopeptidases have proven to be ineffective (28).

## 3 Immunological biomarkers

### 3.1 Donor-derived cell-free deoxyribonucleic acid

Donor-derived cell-free deoxyribonucleic acid (dd-cfDNA) has been proposed as a noninvasive marker for the early detection of rejection before clinical allograft dysfunction (an increase in serum creatinine). Cell-free DNA (cfDNA) is a DNA fragment released from cells with a fast turnover, making it a useful tool for real-time monitoring of allograft damage. In KT recipients, the total cf-DNA in blood can be derived from the cells of the host and donor (allograft), and the differentiation between the cf-DNA from the allograft (donor cells) or recipient cells (host cells) is essential for determining allograft dysfunction. Large quantities of donor cells are found in recipients with graft injury and/or rejection caused by cell death. Similar to the clearance of serum creatinine, the clearance of dd-cfDNA from an individual’s body is comparable to that of cell-free DNA; however, additional study is required. In the circulation, cell-free DNA has a half-life of 16 minutes to 2.5 hours (29, 30). The DNase I enzyme present inside the liver and spleen can cause the entry of cell-free DNA and breakdown by the macrophages there (31). Cell-free DNA can also be excreted via the urine.

The blood level of dd-cfDNA is reported as the percentage of dd-cfDNA to the total cf-DNA, and its usefulness has been explored in several publications. In uncomplicated KT, high blood dd-cfDNA levels are encountered, with a median value of approximately 20% immediately (within hours) after renal engraftment and rapidly decreases on the first postoperative day to approximately 5% and then subsequently to below 1% (32). The level of dd-cfDNA depends on cell lysis (cell damage) (33) from any causes, including inflammation, infection, drug toxicity (calcineurin inhibitors), and disease recurrence. Due to its rapid change, dd-cfDNA can be used to obtain an immediate diagnosis of posttransplant rejection; however, the reported efficacy has varied among different studies (34–37). Sigdel et al. (35) demonstrate a new dd-cfDNA approach that employs a next generation sequencing (NGS) assay with single nucleotide polymorphisms (SNP)-based massively multiplex polymerase chain reaction (mmPCR) in a single-center retrospective analysis. The researchers examine 300 plasma samples acquired from 193 KT patients, including those with routine biopsies. The 217 biopsy-matched plasma samples from 193 KT patients, including 38 active rejection, 72 borderline TCMR rejection, 82 stable allografts, and 25 patients with other damages. Then, mmPCR is used to target 13,392 SNPs in dd-cfDNA. The test is able to distinguish acute allograft rejection (both AMR and TCMR) from non-rejection with an area under the curve (AUC) for the receiver-operator characteristic (ROC) curve (AUROC) curve of 0.87 with 88.7% sensitivity, 72.6% specificity, negative predictive value (NPV) 95.1%, and positive predictive value (PPV) of 51.9% and a stated cutoff of 1%. Unlike other dd-cfDNA technologies, the test is able to differentiate among TCMR, AMR and non-rejection causes (toxic damage or viral infection). Technical advancements enable a highly sophisticated mmPCR method allowing the use of over 13,000 SNP markers (35).

According to a meta-analysis, the sensitivity for AMR diagnosis is high at a fractional threshold of 1%, but less sensitive for TCMR, which generally needs a concentration higher than a 1% threshold, especially if the rejection is more severe than Banff 1B (38). With a cutoff of 0.69-1% for a positive test, most studies with commercially available dd-cfDNA assays demonstrate an AUC at 0.71-0.85, with a sensitivity and specificity of 45-89% and 69-85%, respectively, and a positive and negative predictive value of 52-77% and 66-95%, respectively, when compared with renal pathology, depending on the pretest probability of rejection (39).

Notably, most of the current studies on dd-cfDNA are *ad hoc* tests on patients who probably have a high pre-test risk of rejection. Categorization of blood dd-cfDNA into high (>1%) (35 cases), moderate (0.5-1%) (43 cases), and low (0.5%) (239 cases) among patients at 1-48 months post-transplantation revealed allograft rejection (biopsy within 2 months of dd-cfDNA measurement) in 24 of 62 cases (20%) among patients with moderate or high dd-cfDNA levels (40). The rejection was mostly demonstrated in patients with high (6 in 25 cases; 17%) and moderate dd-cfDNA (5 in 43 cases; 12%) when compared with the low level (13 in 239 cases; 5%) with no difference in the 1.6-year short-term graft outcomes using estimated glomerular filtration rate (eGFR) and *de novo* donor-specific antibodies (DSAs) (40). Most patients with high dd-cfDNA without allograft rejection remain stable without eGFR decline or graft loss (40). By contrast, a recent large multicenter study with approximately 1,100 kidney transplant patients indicated that patients with dd-cfDNA >0.5% had a greater risk of eGFR decline over 3 years and increased *de novo* DSA after follow-up (41).

A strong correlation is evident between high dd-cfDNA (>1%) and subclinical AMR using the Molecular Microscope Diagnostic System (MMDx; molecular tissue gene expression), but not by histopathology, among sensitized recipients (high risk of rejection), as indicated by DSAs, flow crossmatch at transplant, or documented non-adherence medication (42). These findings are also supported by the multicenter Trifecta trial (43). Likewise, Huang and colleagues (44) demonstrated that dd-cfDNA discriminated KT recipients with AMR (median 1.35%, interquartile range (IQR) 1.10% to 1.90%) from those without AMR (median 0.38%, IQR 0.26% to 1.10%), *p <*0.001. Interestingly, dd-cfDNA could not discriminate KT recipients with TCMR from those without rejection (44). A study by Whitlam et al. (45) provides further support, as 61 KT recipients with AMR showed receiver-operator characteristic AUC for graft-derived cfDNA concentration and graft fraction that were predictive of AMR (AUC = 0.91 (95% confidence interval (CI) 0.82 to 0.98) and 0.89 (95% CI 0.79 to 0.98). Again, both measures failed to diagnose borderline or type 1A TCMR (45).

High-normal dd-cfDNA (> 0.5%) can also identify individuals with borderline TCMR 1A histology who are likely to experience deteriorating kidney function (37). Indeed, the majority of patients with high dd-cfDNA and retained allograft function remained stable throughout the study without deterioration of function or graft loss (40). These publications support the preliminary use of dd-cfDNA as a screening test for renal biopsy and for categorizing rejection grading. Nevertheless, KT recipients with high DSA levels, BK poliomavirus (BKV) nephropathy, urinary traction infections, acute tubular necrosis, and post-renal allograft biopsy may also show increases in their dd-cfDNA levels (34, 46). Notably, absolute dd-cfDNA quantification in copies/mL might be more effective than the dd-cfDNA level as the percentage of total cf-DNA for discriminating allograft rejection (36). More studies would be interesting.

In summary, dd-cfDNA is a robust biomarker for the diagnosis of renal allograft rejection. Although dd-cfDNA alone cannot replace renal biopsy, it does provide a noninvasive way of identifying the potential causes of allograft failure in certain recipients, thereby enhancing the ability to predict long-term renal allograft outcomes. Increases in several regular biomarkers, including creatinine, proteinuria, and/or newly increased DSAs, are now indications for further dd-cfDNA tests (47). A routine cross-sectional dd-cfDNA testing of patients with a low pretest chance of rejection might be beneficial, and high dd-cfDNA levels are more common in DSA-positive recipients, highlighting the usefulness of dd-cfDNA in monitoring highly sensitized individuals (48). With the introduction of Allosure^®^ and other comparable tests, dd-cfDNA is already being used as a supporting tool for diagnosis and therapy in clinical practice. The effects of repeated dd-cfDNA surveillance in kidney transplant recipients are currently being assessed in two prospective studies (The Ongoing Kidney Allograft Outcomes Registry (KOAR; NCT03984747), and The Prospera Kidney Transplant ACTIVE Rejection Assessment Registry (PROACTIVE; NCT03984747)

### 3.2 Transcriptomics

Several difficulties arise when attempting renal allograft rejection classification from kidney histology, including a lack of tissue, poor repeatability, and a dearth of well-trained pathologists. For this reason, transcriptome analysis has been the most highly feasible candidate technique for overcoming these limitations, as indicated by the use of C4d (49) and AMR-specific molecular panels (50, 51) for AMR diagnosis. Currently, the Molecular Microscope Diagnostic System (MMDx) is the gold standard for transcriptome analysis of kidney transplantation for AMR and TCMR (52, 53) with the identified key cellular pathways that contribute to rejection. However, many challenges remain in translating molecular diagnostics into clinical practice, including a large number of redundant gene sets that raise a need for standardization of various molecular diagnostic panels on gene analysis (e.g., microarrays and quantitative real-time polymerase chain reaction [qRT-PCR]), as well as an ongoing debate on rejection gene sets between AMR and TCMR (13).

Unlike the microarray gene-based MMDx platform, the NanoString nCounter platform needs only 100 ng of mRNA from formalin-fixed paraffin-embedded (FFPE) biopsies, without a requirement for a biopsy core, to detect mRNA target molecules within two days, allowing large-scale transcriptomic results from biopsy samples (54). The Banff Molecular Diagnostics Working Group developed molecular consensus gene sets for TCMR and AMR in 2015 (55) and proposed several molecular panels in 2017 (13). It subsequently launched the commercially available Banff-Human Organ Transplant (B-HOT) panel for transplantation in several organs (kidney, lung, heart, and liver) in 2019 without centralized molecular profiling (56). The incorporation of molecular pathology into clinical practice may use NanoString technology with the B-HOT panel for better diagnosis, categorization, and normalization, as demonstrated by the different gene expressions observed between no rejection versus AMR and TCMR (57).

Using the most predictive genes from the B-HOT and Element analysis, regression models based on the two least absolute shrinkage and selection operators are being developed to classify biopsies as AMR versus no AMR (57). These classifications include borderline rejection, TCMR, or no rejection, with a receiver-operating characteristic area under the curves (AUC) of 0.994 and 0.894, sensitivity of 0.821 and 0.480, and specificity of 1.00 and 0.979 during cross-validation compared with the gold standard renal biopsy (57). In addition, principal component analysis (PCA) of the microarray gene sets can identify the main categories of renal diagnosis and a comparable relationship between pathological diagnosis and molecular sets (58). As a result, non-chronic antibody-mediated rejection with high expression of endothelial genes can be detected by PC clustering with cell type analysis that is also able to reveal differences in genes from B-cells and plasma cells (58).

In addition, there are several tests that measure immunological activity by looking at the gene expression of circulating immune cells. A widely integrated gene expression profile (GEP) assay is AlloMap, which has been made available as a monitoring tool for heart transplant recipients since 2005 (59) with a high negative predictive value (NPV). However, immune system gene expression profiling in KT has been difficult to use as a consistently accurate and repeatable indicator of renal allograft rejection because the data remains controversial (9, 60–62). A most recent study from Akalin et al. (63) demonstrates the validation of a blood GEP developed to differentiate immune quiescence from both TCMR and AMR. On the basis of 56 peripheral blood samples, a five-gene classifier (DCAF12, MARCH8, FLT3, IL1R2, and PDCD1) is created and validated on two separate sample sets outside of the training cohort. The main validation set includes 18 rejection examples—7 TCMR, 10 AMR, and one mixed rejection—and 98 quiescence samples. The second validation set has 11 rejection samples—7 TCMR, 2 AMR, and 2 mixed rejection—and eight quiescence samples. Interestingly, quiescence and rejection are distinguished significantly by AlloMap Kidney classifier scores in the primary validation set (median, 9.49; IQR, 7.68-11.53 and 11.25-15.28, respectively). The medians in the second validation set are similar to those in the first validation set, although the cohorts are significantly different (*p* =0.03). The primary validation’s AUC for separating rejection from quiescence is 0.786, and the secondary validation’s AUC is 0.800 (63). Thus, blood GEP and dd-cfDNA contribute independent signals and inform on different aspects of allograft rejection.

On the other hand, the Kidney Solid Organ Response Test (kSORT) is a microarray-based assay designed to identify recipients at high risk for acute rejection (64) using quantitative polymerase chain reaction (PCR) to measure the relative mRNA expression levels of 17 genes that are associated with acute renal allograft rejection or leukocyte trafficking in peripheral blood. An algorithm based on correlation is then used to generate risk scores and classify patients as having a high, medium, or uncertain risk of acute rejection. The kSORT assay is initially evaluated in a large multicenter study of 436 adult and pediatric kidney transplant recipients (Assessment of Acute Rejection in Renal Transplantation [AART]) with paired peripheral blood samples and kidney allograft biopsies (performed for allograft dysfunction or as part of a clinical protocol) using a case-control study design of selected recipients (64). With a sensitivity and specificity of 92% and 93%, respectively, the kSORT assay is able to identify patients at high risk of either TCMR or AMR. In addition, kSORT is able to identify subclinical rejection in 75% of biopsies and clinical rejection in over 60% of samples collected within three months prior to the diagnosis of biopsy-confirmed acute renal allograft rejection. Nonetheless, the test fails to differentiate between acute TCMR and AMR.

Moreover, the TruGraf^®^ v1 assay is a DNA microarray-based gene expression blood test that is developed as an alternative to surveillance biopsies to rule out subclinical rejection in recipients with sustained graft function (65). Blood samples coupled with protocol biopsies from prevalent cohorts are utilized for the entirety of the discovery and external validation of the TruGraf^®^ test. However, the performance of the test in recipients with renal allograft dysfunction has not been evaluated and must be studied further. Interestingly, combining the TruGraf^®^ assay with dd-cfDNA enhances the detection of subclinical renal allograft rejection (66). Of note, by using multivariable logistic regression, the AUC is 0.81, which is substantially greater than the gene expression profile (*p* <0.001) or dd-cfDNA alone (*p* =0.006). Notably, when cases are divided according to rejection type, the gene expression profile is significantly better at detecting TCMR (AUC 0.80 versus 0.62; *p* =0.001), whereas the dd-cfDNA is significantly better at detecting AMR (AUC 0.84 versus 0.71; *p* =0.003) (66).

To sum up, at present, transcriptomic analysis is revealing the possible molecular mechanisms that might improve outcomes and be useful as precision diagnostic indicators in renal transplantation.

### 3.3 MicroRNAs

MicroRNAs (miRs) are a class of short, noncoding RNAs that can regulate gene expression (57). They can be detected by several different methods, including qRT-PCR, microarray, and next-generation sequencing analysis (global miR profiling) (67) in the blood (cells), serum/plasma, and urine (68, 69). Ischemic reperfusion injury during KT increases urine miR-146a content to higher levels in renal transplant recipients implanted from deceased donors than from living donors (70). Acute TCMR increases miR-223 and miR-142–3p in allografts and in peripheral blood mononuclear cells (PBMCs) of recipients (71). Patients with TCMR demonstrate higher miR-223, miR-10a (72), miR-99a, and miR-100 levels in blood samples (73), but lower levels of miR-99a expression in kidney allografts (74, 75), implying a possible difference in miR levels between renal tissue and blood samples. Interestingly, multivariable logistic regression analysis of a panel of blood miRs (miR-15b, miR-16, miR103a, miR106a, and miR-107) was able to differentiate acute vascular rejection (Banff II–III) from stable graft function (76). In acute TCMR, urinary miR-10a is upregulated, while miR-10b and miR-210 are downregulated. The urinary level of miR-210 (a cellular aging biomarker) is correlated with the severity of biopsy-proven rejection, but with low specificity and sensitivity, unfortunately (69). Increased levels of miR-142–5p are reported in the PBMCs of recipients with chronic, but not acute, AMR (77) and with acute TCMR (71, 74). Interestingly, alteration of miR levels between pre- and post-renal allograft rejection has been reported by Millán and colleague study group (78). As such, urinary levels of miR-142-3p and miR-155-5p significantly increase, while miR-210-3p decrease in allograft rejection. The miR-155-5p at the threshold values of 0.51 demonstrates sensitivity and specificity at 85% and 86%, respectively, and the analyses of receiver operating characteristic (AUC) effectively differentiate the recipients with versus without allograft rejection (AUC = 0.875; *p* =0.046) (78). Also, there is a good correlation between miR-155-5p and glomerular filtration rate or renal allograft restoration (78).

Additionally, the content of miR-211, miR-204, and miR-142–3p in the urine exosomes of patients with biopsy-proven IF/TA show a correlation between miRs in urine and renal tissue (79). Downregulation of miR-200b, miR-375, and miR-193b and upregulation of miR-423–5p and miR-345 are also detected in the urine of patients with IF/TA (one-year follow-up) without the association between miR-200b expression and proteinuria (68). Downregulation of miR-200b (80) and downregulation of miR-21 are observed in plasma from patients with IF/TA (81).

In summary, many miRs have been proposed as biomarkers for renal allograft dysfunction due to miR stability; however, assessment using receiver-operator characteristic areas under the curves (sensitivity and specificity) is limited. Nevertheless, a five-miR panel is able to distinguish T cell–mediated vascular rejection from stable graft function following kidney transplantation (76), implying possible benefits of combined miR (panels). MiRs from allograft biopsy tissue provide greater accuracy for rejection diagnosis, suggesting that tissue-derived miRs may have the potential to substitute for histology. More studies are warranted.

### 3.4 Extracellular vesicles (EVs) and exosomes

Extracellular vesicles (EVs) are bilayer lipid membranes released by all cells in the body and can include exosomes, microvesicles (MVs), ectosomes, oncosomes, and apoptotic bodies. In general, the term “EV” seems to be a generic label for a “secreted vesicle” (82). The EVs in body fluids operate as carriers in signal transmission between cells for the regulation of immunological responses, inflammation, and other cell activities (83, 84). Because all cells can generate EVs, the EVs in urine should be correlated with the cells with direct urine contact (e.g., the urinary epithelium, endothelium, and immune cells). By contrast, the source of cells that produce EVs in blood could be more difficult to determine. The determination of EVs from urine requires strict normalization, and normalization by the duration of urine collection (time normalization), especially 24-hour urine, seems to be mostly appropriate; however, unfortunately, the correlation observed between EVs in urine and other normalization biomarkers (creatinine, total proteins, number of EVs) remains inconclusive (85). The duration of urine in the bladder before urine collection might also alter the EVs in the urine sample, because bladder cells can also produce EVs, and those EVs could be altered by urine characteristics (pH, concentration, and excreted substances) (85). Nevertheless, EVs from both blood and urine are being extensively studied for biomarkers.

Among all the EV types, exosomes were observed for the first time in a multivesicular endocytic compartment in 1983 by Harding et al. (86). Since then, these EVs have undergone the most extensive exploration. Exosomes are 40-100 nm in diameter (82) and are formed as lipid bilayers that can protect several molecules inside. For example, several RNA types, including miRs, long noncoding RNAs (lncRNAs), small nuclear RNAs (snRNAs), and circular RNAs (circRNAs), are found in EVs and can be used as biomarkers (87). Current omics technology, including transcriptomics, proteomics, and metabolomics, is now used for the genetic association analysis of expression quantitative trait loci (eQTL), protein quantitative trait loci (pQTL), and methylation quantitative trait loci (mQTL) (88). This has made possible the expanded use of exosomes and EVs for locating potential sites in allografts that produce EVs (89). Despite the large number of EVs in the plasma (roughly 10^2^–1.0^13^ vesicles per mL) (90), the tiny size, limited contents, and possible difference in contents inside each particle (referred to as “liquid biopsy”) are limitations for the use of EVs as biomarkers. However, next-generation sequencing (NGS) and mass spectrometry can now amplify and detect the molecules within the vesicles or the intra-vesicular contents of EVs and have revealed several interesting aspects of EVs.

One example is the profile of urinary EVs from living-donor renal transplantation, which demonstrates that the EVs are derived from the nephron (glomeruli and other parts; descending limb of Henle’s loop, the collecting tubules, etc.), epithelium, and endothelium (91). This categorization is established by the detection of several molecules, such as megalin, aquaporin (AQP), podocalyxin (PODXL), ion cotransporters, synaptotagmin 17 (SYT17), CD3, and CD133, which are expressed only at specific sites and might therefore be useful as biomarkers (92–94). Increases in these molecules in EVs from urine or blood mostly indicate that some damage has occurred to renal allografts.

Interestingly, the EV molecules related to epithelial cell differentiation seem to be upregulated in TCMR, while proteins of acute inflammation or antigen presentation are more related to AMR (95). Likewise, the levels of the sodium-chloride cotransporter (NCC) and Na-K-Cl cotransporter (NKCC2), the transporters commonly found in renal tubular cells, are higher in the EVs (exosomes) from patients treated with calcineurin inhibitors (CNIs; drugs with tubular toxicity) than with non-CNI regimens (96, 97). Similarly, miRNA-enriched EVs are reported in patients who experience long ischemic times during transplantation (98), implying that EVs might be directly related to ischemic mechanisms through the delivery of miRs and other molecules from one cell to others (99, 100).

In 2017, a landmark study by Park and colleagues reports the use of EVs in renal allograft rejection as T cell-derived EVs in urine might indicate renal tubular T cells infiltration during TCMR (101). Thus, an EVs-based diagnostic platform recognizing T cell-derived urinary EVs (uEVs), refer to as iKEA (integrated kidney exosome assay), is mentioned as TCMR biomarker. As such, CD3 is used to identify T cell-derived uEVs and the CD3-based iKEA demonstrates diagnostic accuracy of 91.1% in a discovery group of 30 recipients and 83.7% in a validation cohort of 14 recipients in subsequent clinical trials (101). Accordingly, iKEA might be a powerful noninvasive serial monitoring in kidney transplant recipients for better long-term renal allograft function. A subsequent well-design, large cohort study from El Fekih et al. established the rejection signatures using approximately 200 samples of the matched urinary exosomal mRNAs with the tissue of renal allograft biopsy for a powerful noninvasive liquid biopsy to identify renal allograft rejection (102). For the diagnosis of all-cause renal allograft rejection, the AUC of renal biopsy is 0.93 (95% CI, 0.87 to 0.98), while the AUCof eGFR is 0.57 (95% CI, 0.49 to 0.65). In parallel, the AUC of urinary exosome-based signature is 0.87 (95% CI 0.76 to 0.97) with positive and negative predictive values at 86.2% and 93.3%, respectively. Additionally, the exosome-based signature distinguishes recipients with TCMR from those with AMR with positive and negative predictive values at 77.8% and 90.6%, respectively (102). Despite a lower AUC than the gold standard renal allograft biopsy, the urine-based exosome measurement is noninvasive and can be frequently measured.

On the other hand, an elevation of EV numbers containing CD31 (glycosylated immunoglobulin-like membrane receptor of leucocytes, platelets, and endothelial cells) or CD81 (Tetraspanin; expressed in several cells except for erythrocytes, platelets, and neutrophils) is correlated with the length of cold ischemia, increased donor age, and reduced renal allograft blood flow (103). This suggests that the removal of EVs in KT recipients who experience long cold ischemic times before renal engraftment might be beneficial (104). The EVs may also transmit viruses through *en bloc* transmission of several viral genomes, which could modulate viral fitness and protect viruses within the lipid membrane (105). Viral particles in EVs might also dilute the physiologic contents and interfere with normal cell–cell communication (106). One virus, the BK polyomavirus (BKV), is an important cause of renal allograft failure (107). Its presence in exosomes could encode the host’s miRs and downregulate some host genes required for viral evasion processes (108), as elevated levels of miR-B1-5p and miR-B1-3p in urinary exosomes indicate possible BKV infection (109, 110).

Several challenges remain for the use of exosomes or EVs as biomarkers. These include methods for the purification and isolation of EVs (or exosomes) that preserve their integrity (111), the normalization, and the time-consuming procedure for 24-hour urine collection. Regarding the therapeutic aspects, EVs also represent possible vehicles for delivering therapeutic molecules to specific target cells (112), while the removal of EV-mediated ischemic responses might improve the long-term outcomes of KT (104). More clinical trials involving several candidates undergoing pre-clinical studies will be very interesting.

### 3.5 Urine and circulating chemokines

Inflammation is a response to cell damage, and detection of inflammation in renal allografts, especially with other biomarkers or clinical characteristics, possibly indicates allograft rejection. For example, urinary CXCL9 and CXCL10 are both increased in AMR and TCMR compared with patients with no rejection (113–115), elevated urinary CXCL10 predicts rejection (78), and treatment of allograft rejection reduces CXCL10 (78, 113, 116). However, combining CXCL9 with CXCL10 does not enhance the prediction ability compared with each molecule alone (114, 117). As an indicator of allograft rejection, urinary CXCL9 demonstrates sensitivity and specificity of 58-86% and 64-80%, respectively, while the values for CXCL10 are 59-84% and 76-90%, respectively (78, 113–115, 117). However, urinary CXCL10 seems to be associated with tubulointerstitial inflammation and peritubular capillaritis, rather than glomerulitis or isolated vascular inflammation (118) and urinary CXCL10, but not CXCL9, correlates with subclinical rejection (AUC 0.64; 95% CI, 0.55-0.73) (116). Both urinary CXCL9 and CXCL10 distinguish rejection from other non-rejection causes of graft dysfunction, with AUCs of 0.72 and 0.74, respectively (116). The urine CXCL10/creatinine ratio, together with the mean fluorescence intensity (MFI) of DSAs, predicts AMR and graft loss better than the DSA MFI alone, with a net reclassification increase of 73% (119). Nevertheless, urinary CXCL10 is not specific for rejection, although it is a good indicator of renal inflammation, as urinary CXCL10 is also elevated to similar levels in patients with BK viremia and in patients with tubulitis from rejection (113, 114). Interestingly, urinary CXCL10 is not increased in cytomegalovirus (CMV)-infected subjects (118), perhaps because of the greater genitourinary specificity of the BK virus compared with CMV. Urine CXCL9 and CXCL10 are also increased in patients with isolated leukocyturia and urinary tract infections (120) and leukocyturia with increased CXCL10 demonstrates more severe inflammation than leukocyturia alone (113). Notably, the levels of urinary CXCL9 and CXCL10 in both absolute terms and after adjustment to urine creatinine (urine creatinine normalization) are useful.

Urinary chemokines are enhanced before rejection becomes clinically apparent, implying that they are good candidates for screening tests (116, 121). Recipients with high urine CXCL10 levels have been divided into renal biopsy or regular surveillance in an ongoing multicenter trial (NCT 03206801). This trial could provide an opportunity to determine whether urinary chemokine levels, when considered alongside histologic variables, can improve the prediction of renal allograft outcomes. A test using urinary chemokines as KT biomarkers will be interesting. Recently, the Barcelona Consensus on Biomarker-Based Immunosuppressive Drugs Management in Solid Organ Transplantation has a preliminary proposal for using urinary chemokine CXCL9 and CXCL10 to guide and individualize immunosuppressive regimens, predict acute and chronic TCMR and AMR, and may be a useful tool for risk stratifying recipients. However, the standard immunoassay platforms are needed (122).

Circulating or plasmatic chemokines, CXCL10 is also a promising biomarker for renal allograft rejection determination. Due to the prevalence of clinical confounding factors, the utility of serum CXCL10 as a potential biomarker for assessing the risk of rejection remains controversial (123, 124). High serum CXCL10 during the pre-transplantation period is associated with long-term graft loss after kidney transplantation (123). As such, Xu et al. (125) demonstrate that serum CXCL10 measured on the fourth and seventh days after kidney transplantation are substantially higher in recipients with acute renal allograft rejection than those without rejection. The most recent study conducted in 28 recipients experienced rejection (14 TCMR cases and 14 recipients with AMR), 8 cases of subclinical rejection, 13 BKV infection, and 16 cases of CMV. Accordingly, in comparison with non-rejection, pre-transplantation circulating CXCL10 is significantly higher in TCMR and AMR. In post-transplantation, increased circulating CXCL10 is demonstrated in TCMR, AMR, and subclinical rejection. All CMV infected recipients show elevated circulating CXCL10 above the rejection threshold, whereas 80% of BKV infected recipients have CXCL10 concentration approximately at 100 pg/mL (126). Indeed, circulating CXCL10 can be used for pre-transplanted stratification and the selection of immunosuppressive regimens following the risk of rejection according to CXCL10 levels. However, BKV and CMV infection must be firstly excluded when using CXCL10 as a rejection biomarker (126).

On the other hand, urinary concentrations of neutrophil gelatinase-associated lipocalin (NGAL), during the early post-transplantation period, have been extensively examined as a predictor of delayed graft function in kidney transplantation (127, 128). Likewise, urine NGAL is demonstrated as a predictor of acute kidney injury in the later period after transplantation (129, 130) and an indicator of allograft loss after acute kidney injury (131). However, the diagnostic utility of NGAL in kidney transplant patients after the first year of transplantation with chronic processes of injury (a steadily deteriorated renal function) is demonstrated by only a few studies (132, 133). Additionally, the difference in urine NGAL assays in various studies makes it difficult for comparison and to propose the cut-off values using data from different studies. A recent study by Kielar et al. (134) demonstrates 2 folds higher urinary NGAL after 1-year post-transplantation in recipients with at least a 10% reduction in eGFR compared to those with stable or improved function of the transplanted kidney. Independent of baseline eGFR and albuminuria, baseline NGAL strongly predicts the relative and absolute changes in eGFR as well as the mean eGFR during the follow-up. Furthermore, high urine NGAL levels in clinically stable kidney transplant recipients after the first year may be interpreted as a warning sign, prompting a search for transitory or chronic causes of graft failure or urinary tract infection (134).

While urinary NGAL might be associated with delayed graft function (127), the relationship between urinary kidney injury molecular-1 (uKIM-1) and renal allograft is not clear (135). As such, recipients with lower KIM-1 in the first week post-transplantation take a longer time to stabilize their renal function compared to the cases with normal uKIM-1. In addition, a prospective cohort study by Zhu et al. (136), in 160 recipients scheduled for kidney transplantation, is conducted to evaluate the predictive power of uKIM-1 for renal allograft prognosis. They discover that recipients with higher uKIM-1 levels on the first day after transplantation had a 23.5% higher risk of developing functioning delayed graft function and a 27.3% higher chance of having a longer renal allograft survival. Hence, it is possible that KIM-1 has a potential role in post-transplant renoprotection (137, 138).

### 3.6 Nucleosomes

The smallest structural component of chromatin is called a nucleosome and usually consists of 8 histone proteins and 146 DNA base pairs (139). The histone-encased DNA plays a crucial role in the epigenetic control of gene expression by modifying the “tail” regions of histones by methylation, acetylation, ubiquitination, and phosphorylation (140). After cell death, nucleosomes are released into the blood, modified by some enzymes, and are then referred to as “circulating cell-free nucleosomes” (CCFN) (141). The epigenetic signature of histones (histone alterations) in CCFNs might be able to differentiate between regular versus pathological cell deaths, as mentioned in cancer studies (142). For example, the addition of DNA modification (5-methylcytosine) and histone modifications (H2AZ, H2A1.1, and H3K4Me2) increased the diagnostic values of carbohydrate antigen (CA) 19-9, a conventional cancer biomarker, in pancreatic malignancy (143). Likewise, increases in nucleosomes with histone alterations are observed in acute renal allograft rejection (144). Indeed, the levels of CCFNs containing citrullinated histone H3 (Cit-H3), a biomarker of neutrophil extracellular traps (NETs) (145–147) important in AMR (118), are increased within several hours after AMR and can be detected using a modest quantity of sample (10 μL) (143). However, serial readings of histone-modified CCFN might be necessary, as the levels may fluctuate in the setting of acute renal allograft rejection. Notably, total nucleosome concentrations (absolute total CCFNs, regardless of histone modification) are only an indicator of cell damage, while CCFNs with specific nucleosome modifications can determine the cause of cell damage and possibly serve as useful markers for renal allograft rejection. More studies on this topic will be interesting.

## 4 The utility of molecular immune monitoring for renal allograft rejection in clinical practice

### 4.1 PROS and CONS

The most advantage of molecular immune monitoring for renal allograft rejection is the superior sensitivity and specificity to the conventional markers (serum creatinine, eGFR, proteinuria, and DSAs) which can reduce unnecessary invasive renal allograft biopsy (148). With conventional markers, detection of subclinical changes is difficult due to the lower sensitivity. Although serum creatinine at one-year post-transplantation reflects long-term renal allograft outcome (149), an individual serum creatinine level is neither sensitive nor specific for early renal allograft injury, particularly compared to urine chemokines (114). Likewise, both albuminuria and proteinuria are nonspecific markers of renal allograft injury without a demonstrable association with renal allograft pathology (150, 151). Although current data support the use of *de novo* DSAs post-transplantation which is associated with decreased renal allograft survival (23, 152), the utilization of DSAs as a noninvasive diagnosis of AMR or a predictor for the long-term renal allograft outcomes has not been clearly elucidated (23). As such, innovative strategies (molecular immune monitoring methods) have been developed to overcome these limitations of the existing biomarkers. Most noninvasive molecular immune monitoring tools, including miR, gene expression, or protein level detection of molecular markers, have been proposed using the easily accessible biologic fluids (blood, serum, plasma, or urine) through a wide spectrum of platforms, mostly for frequent assessment of recipient’s immune status. However, the translation and validation of these discoveries and their implementation into standard transplantation clinical practice remain challenging. More large, prospective, interventional clinical trials are robustly needed to demonstrate the use of these molecular immune monitoring biomarkers for the improvement of renal allograft outcomes. In general, significant limitations of using these novel noninvasive molecular markers in clinical practice are regulatory issues, reimbursement from the Centers for Medicare and Medicaid Service, generalizability, cost, interpretation of the test, and, most importantly, the identification of beneficial populations compared with the conventional standard-of-care surveillance (153).

### 4.2 Combined molecular immune monitoring and the clinical parameters as a predictive score for renal allograft rejection

Due to the complexity and variability of immune responses, a panel of biomarkers (such as chemokines, DSAs, dd-cfDNA, and several miRs) might be more powerful than a single indicator for the prediction and diagnosis of renal allograft rejection and the differentiation between TCMR and AMR. For example, the Common Rejection Module that consists of 11 genes might be overexpressed in the biopsy samples from various solid organ transplants, including renal allograft rejection (154). Additionally, the urinary gene expression-based score (mRNA of these 11 genes) using urine from 150 renal transplant recipients with concurrent renal biopsies, including 43 stable biopsies, 45 acute rejections (TCMR or AMR or mixed), 19 ambiguous rejections, and 43 BKV, demonstrates 95% and 98% sensitivity and specificity, respectively, for the diagnosis of acute rejection (155). The sensitivity of the urinary gene expression-based score for diagnosis of acute renal rejection is reduced to 87.1% with an addition of the cases with ambiguous renal rejection into the stable biopsy and is decreased to 77% sensitivity with an addition of BKV nephropathy cases, with an unchanged specificity (155). Then, the urinary gene expression-based score may be useful for the non-invasive monitoring of acute renal allograft rejection.

Indeed, the addition of potential confounding cases (such as urinary tract infection and BK virus reactivation) in the stable biopsy as “a diagnostic multi-parametric model” improves the performance of the biomarkers (120). As such, a model with the combination of eight parameters (recipient age, gender, eGFR, DSA, signs of urinary tract infection, blood BKV viral load, urine CXCL9, and CXCL10) is able to diagnose acute renal allograft rejection with high accuracy (AUC: 0.85, 0.80–0.89). These results are paving the way for future studies using the combining urinary biomarkers with clinical characteristics to achieve the highest clinical relevance and provide targeted therapy for KT recipients (120). Recently, a research group from the University of California San Francisco demonstrates another comprehensive noninvasive tool for diagnosing and predicting renal allograft rejection (156). They explore the performance of target markers in a Kidney Injury Test assay for chronic kidney disease (CKD) staging in the native and non-transplanted kidney (157) and develop a Q-Score from these data for the detection of acute renal allograft rejection. Based on measurements of six urinary DNA, protein, and metabolic biomarkers, a noninvasive, spot urine-based diagnostic assay is proposed. On a cohort of 601 distinct urine samples with kidney injury (both native kidneys and renal allografts), the urinary composite score enables the diagnosis of acute renal allograft rejection, with an AUC of 0.99 for the receiver-operator characteristic (ROC) curve. Interestingly, the clinical utility of the assay can predict acute renal allograft rejection better than an increased serum creatinine resulting in an earlier rejection diagnosis than the current standard-of-care tests (156).

In summary, the use of a combination of multiple variables with mathematical approaches to calculating rejection probability, but not using only biomarkers of “graft functional impairment” alone might be very useful for an early diagnosis of rejection and might also be helpful for the selection of immunosuppressive protocols. Additionally, the rapid and routine monitoring of renal allografts is possibly enabled by the noninvasive assays, especially with sensitive and quantitative methods.

## 5 Future directions

While the establishment of a worldwide consensus framework (i.e., the Banff criteria) is still ongoing, a great deal of progress has been made in the field of the diagnostic evaluations of allograft pathology. In the foreseeable future, a molecular diagnostic model for renal allograft pathology should show significant steps toward the final development of a decentralized multi-platform compatible system. This could significantly impact clinical practice and outcomes by placing particular emphasis on the complex normalization pipelines required to compare gene expression data generated by different technologies. The creation of this system must integrate the efforts of the whole transplantation community for its validation to ensure that these molecular technologies provide optimal performance. In addition, the continuous updating of diagnostic criteria for renal allograft rejection and related lesions has improved diagnostic accuracy and clinicopathologic correlations, while also helping to clarify the limitations of histology and immunohistology in renal allograft biopsy interpretation. This has highlighted the need for the development of additional diagnostic modalities, including molecular diagnostics.

## 6 Conclusions

New-generation biomarkers in kidney transplantation are a collection of advanced indicators that provide a more comprehensive understanding of the status of a renal allograft. This has enabled the prognosis of the ultimate long-term renal allograft outcomes through the early detection of renal allograft rejection or dysfunction (**Table 1**). Although these biomarkers are now promising, further study is required to establish their therapeutic relevance and to find appropriate procedures for measuring and interpreting the data, especially in kidney transplant recipients. The choice of biomarkers may rely on the specific research topic, the type of accessible sample, and the isolation and analysis procedures employed. Interestingly, the integration of numerous indicators for a complete approach may improve accuracy and provide a bird’s-eye perspective of the condition of kidney allografts in individual recipients.

**Table 1:** Summary of the novel biomarker studies of immunologic monitoring in kidney transplant rejection.

Biomarkers (Commercial Assay)	Sample	N	Primary outcome(s)	Sensitivity (%)	Specificity (%)	PPV (%)	NPV (%)	Study outcomes	Author, Year [References]
Donor-derived cell-free deoxyribonucleic acid (GRADE certainty rating ^a^: MODERATE)									
dd-cfDNA (Allosure)	Plasma	102	Rejection	59	85	61	84	 Differentiation between rejection (TCMR and AMR) versus non-rejection and between AMR versus non-AMR recipients (AUC = 0.74)	Bloom et al, 2017 (34)
dd-cfDNA (Allosure)	Plasma	63	AMR	68	72	74	65	 dd-cfDNA discriminates AMR, but not TCMR, from non-rejection (AUC = 0.71)	Huang et al, 2019 (44)
dd-cfDNA (Prospera)	Plasma	217	AMR	88.7	72.6	52	95	 dd-cfDNA discriminates AMR, and TCMR from non-rejection (AUC = 0.87)	Sigdel et al, 2018 (35)
dd-cfDNA (noncommercial)	Plasma	61	Acute AMR, chronic AMR	AMR: 0.90	AMR: 0.88	60	98	 dd-cfDNA and fraction are predictive of acute AMR (AUC = 0.92, 0.85) and composite diagnosis of AMR (AUC = 0.91, 0.89)	Whitlam et al, 2019 (45)
dd-cfDNA (noncommercial)	Plasma	189	Rejection	73	73			 Recipients with biopsy-proven rejection demonstrate 3.3-folds higher dd-cf-DNA (copies/mL) and 2.0-folds higher dd-cf-DNA (%) than those without rejection.  dd-cfDNA absolute number is higher than dd-cfDNA in % (AUC = 0.73), OR = 7.31 for dd-cfDNA (copies/mL)	Oellerich et al, 2019 (36)
dd-cfDNA	Plasma	19	Rejection, BK polyoma virus nephropathy (BKPyVAN)					 BKPyVAN is associated with a slight increase in dd-cfDNA (median; IQR: 0.38% [0.27%-1.2%] vs. 0.21% [0.12%-0.34%] in non-rejection control recipients.  dd-cfDNA levels are far lower than AMR (1.2% [0.82%-2.5%], but not different from TCMR.	Mayer et al, 2019 (158)
dd-cfDNA	Plasma	79	eGFR, rejection prediction, *de novo* DSA					 Increased dd-cfDNA predicts adverse outcomes as following: a) Recipients with increased dd-cfDNA have decreased eGFR by 8.5% compared with 0% in those with decreased dd-cfDNA b) *de novo* DSA is demonstrated in 40% vs 2.7% of recipients with increased or decreased dd-cfDNA, respectively c) Persistent rejection is developed in 21.4% of cases	Stites et al, 2020 (37)
dd-cfDNA (noncommercial)	Plasma	29	Acute rejection	88	81	64	94	 dd-cfDNA levels discriminate between recipients with biopsy-proven acute rejection (median 5.24%; range 1.00–9.03), recipients without acute rejection (1.50%; 0.41–6.50), and recipients with borderline acute rejection (1.91%; 0.58–5.38).  dd-cfDNA is significantly differences between recipients with versus without acute rejection (AUC = 0.84)	Dauber et al, 2020 (159)
Transcriptome (GRADE certainty rating ^a^: MODERATE)									
Gene expression profile	Plasma	308	Subclinical acute rejection	64	87	61	88	 Gene expression profile of acute rejection predicts subclinical rejection	Friedewald et al, 2019 (160)
Targeted expression assay (TREx)	Plasma	113	Acute rejection at 3 months, renal allograft failure			79%	98%	 TREx predicts subclinical rejection at 3 months in 113 recipients (AUC = 0.830)	Zhang et al, 2019 (61)
Kidney Solid Organ Response Test (kSORT^™^) and enzyme-linked immune absorbent spot (ELISpot)	Plasma	75	Surveillance of recipients with stable renal allograft function					 kSORT^™^ and ELISpot predict subclinical TCMR and subclinical AMR (AUC > 0.85)	Crespo et al, 2017 (161)
TruGraf® gene expression profile	Plasma	Retrospective 192 recipients in 7 transplant centers with a prospective observational study in 45 recipients at 5 transplant centers.	Acute rejection					 TruGraf® affects to physician’s clinical decision in 87.5% of cases  45 recipients’ TruGraf® supported 87% of clinical decisions with 93% of investigators stating that they will use TruGraf® for their clinical practice	First et al, 2019 (162)
11 Common rejection genes	Urine	150 (43 stable renal allograft, 45 acute rejection, 19 borderline pathology, and 42 BK virus nephropathy)	Acute rejection	93.6	97.6			 10 from 11 genes are elevated in acute rejection compared with stable renal allograft function. Of note, Psmb9 and CXCL10 could classify acute rejection from stable renal allograft function as accurately as the 11-gene model  Urinary common rejection model (uCRM) score differentiates AMR from stable renal allograft function (AUC = 0.9886)	Sigdel et al, 2019 (155)
MicroRNAs (GRADE certainty rating ^a^: LOW)									
miR-15B, miR-103A, miR-106A	Plasma	160	TCMR					 miR-15B, miR-103A, and miR-106A discriminate recipients with stable renal allograft function from the recipients with TCMR and urinary tract infection.	Matz et al, 2016 (163)
miR-223-3p, miR-424-3p, miR-145-5p	Plasma	111	TCMR, AMR					 miR-145-5p, miR-223-3p, and miR-424-3p discriminate recipients with stable renal allograft function from TCMR and AMR.	Matz et al, 2018 (164)
miR-142-3p, miR-155-5p, miR-210-3p, CXCL10	Urine	80	Acute rejection	85% 84%	86% 80%			 Increased miR-142-3p, miR-155-5p, CXCL10 and decreased miR-210-3p discriminate recipients with rejection and nonrejection	Millán et al, 2017 (78)
Molecular Microscpoic® Diagnostic System (MMDx™)/microRNA	Renal allograft tissue	519	TCMR, AMR					 The agreement rates between MMDx™ and renal allograft tissue pathology are 76%-77% for TCMR, AMR, and non-rejection  The MMDx™ is correlated with clinical judgment (87%) more than histology (80%).	Halloran et al, 2017 (53)
microRNA	Renal allograft tissue	11 studies	TCMR, AMR, and chronic AMR					 Increased miR-142, miR-155, miR-223 and decreased miR-30, miR-125, miR-204 predict the primary outcomes	Ledeganck et al, 2019 (165)
Extracellular vesicles and exosomes (GRADE certainty rating ^a^: MODERATE)									
Exosomes	Serum	213 kidney transplant alone recipients, and 14 kidney-pancreas transplant recipients	Acute rejection is identified as CD31^+^/CD42b^−^ microparticles and quantified by fluorescence-activated cell scanning					 Increased circulating exosomes levels is associated with acute rejection.  Circulating exosomes are rapidly decreased after treatment for rejection in recipients with negative peritubular capillaritis C4d, but the decrease is slower in those with positive peritubular capillaritis C4d.	Qamri et al, 2014 (166)
	Urine (using LC-MS/MS method)	30	Acute rejection					 Eleven urine exosomal proteins are more abundant in urine samples from recipients with acute rejection.  3 out 11 of urine exosomal proteins are exclusive for the exosomal fraction.  Exosomal acute rejection-specific biomarkers are also detected in unfractionated whole urine.	Sigdel et al, 2015 (167)
	Urine	Discovery phase (n = 30): 15 non-rejection recipients, 15 acute rejection, 3 chronic AMR, and 3 BK polyoma virus nephropathy. Validation cohort (n = 14): 7 acute rejection and 7 non-rejection recipients)	Acute rejection by using urine-based platform to detect iKEA					 Significantly higher level of CD3^+^ exosomes among recipients undergoing TCMR, very low CD3^+^ extracellular vesicle levels in BK poliomavirus nephropathy and chronic AMR recipients, supporting the specificity of iKEA for TCMR.	Park et al, 2017 (101)
		64 (18 AMR, 8 TCMR, and 38 non-rejection recipients)	TCMR and AMR by identified as mRNA expression					 Among 21 candidate genes, multiple genes are identified (gp130, CCL4, TNFα, SH2D1B, CAV1, atypical chemokine receptor 1 [Duffy blood group]) whose mRNA transcript levels in plasma exosomes significantly increased among AMR compared with TCMR and/or control recipients.  A gene combination score calculated from 4 genes of gp130, SH2D1B, TNFα, and CCL4 is significantly higher in AMR than TCMR and non-rejection recipients.	Zhang et al, 2017 (168)
	Urine	47 (22 stable renal allograft function, 25 TCMR)	TCMR					 17 proteins are increased in TCMR patients.  Of all candidate biomarkers, tetraspanin-1 and hemopexin are two most significantly higher proteins in TCMR recipients.	Lim et al, 2018 (169)
	Urine and renal allograft tissue	78 (20 normal histology, 19 IF/TA, 17 calcineurin inhibitors toxicity, and 22 chronic active AMR)	Detection of exosomes-Western blot with antibody against SYT17 biopsies -immunohistochemistry with anti-SYT17, anti-STAT3 pY705, and anti-phospho NFκB p65 Ser276 antibodies					 No SYT17 protein is detected in whole-urine samples.  SYT17 proteins are detectable in urinary exosomal fractions and high enrichment of SYT17 in exosomes from urine of chronic active AMR recipients compared to healthy volunteers and individuals in the normal renal allograft histology.  SYT17 protein is expressed strongly in the chronic active AMR recipients compared to other recipient groups.	Takada et al, 2020 (94)
	Urine (At 1-week, 1-month, and 3-month post transplantation	23	Allograft function, immunosuppressive drug levels, and acute rejection by identified miRNA’s expression					 Three overexpressed urinary exo-miRs (miR-146b, miR-155, andmiR-200a) in recipients are negatively correlated with tacrolimus dose.  MiR-200a is positively correlated with proteinuria.	Freitas et al, 2020 (170)
	Urine and renal allograft tissue (for cause biopsy)	175 kidney transplant recipients undergoing for cause biopsy, with 192 urine samples that have matched biopsy specimens are included.	TCMR, AMR					 An exosomal mRNA signature discriminated between biopsy samples from recipients with all-cause rejection and those with non-rejection.  Additional gene signature discriminated recipients with TCMR from those with AMR.	El Fekih et al, 2021 (102)
Chemokines (GRADE certainty rating ^a^: LOW)									
CXCL9, CXCL10	Urine	244	Acute rejection					 CXCL9 and CXCL10 are correlated with total inflammation and microvascular inflammation.  Ratio of CXCL10:SCr and DSA in the improved diagnosis of AMR (AUC = 0.83).	Rabant et al, 2015 (114)
CXCL9	Urine	21	Acute rejection					 CXCL9 predicts acute rejection by a median of 15 days before clinical presentation of acute rejection	Hricik et al, 2015 (121)

a GRADE (Grading of Recommendations, Assessment, Development, and Evaluations) comprises 4 ratings: very low, low, moderate, and high (171). AMR, antibody-mediated rejection; AUC, area under the curve; CXCL, C-terminal amino acid sequence Cystine-X-Cystine motif chemokine ligand; dd-cf-DNA, donor-derived cell-free deoxyribonucleic acid; DSA, donor specific antibodies, eGFR, estimated glomerular filtration rate; IF/TA, interstitial fibrosis and tubular atrophy; iKEA, integrated kidney exosome assay; LC-MS/MS, liquid chromatography−tandem mass spectrometry; NPV, negative predictive value; PPV, positive predictive value; SCr, serum creatinine; TCMR, T cell-mediated rejection

## Author contributions

The followings are the authors’ contribution: conceptualization, WC, OT, and AL. Writing—original draft preparation, WC. Writing—review and editing, WC, OT, AL AT. Funding acquisition, WC; and supervision, AL and AT. All authors contributed to the article and approved the submitted version.
